# Acute and Chronic Middle-Ear Suppuration, with Special Reference to Treatment1Read at the Geneva Medical Society, September, 1904.

**Published:** 1905-04

**Authors:** George F. Cott

**Affiliations:** Clinical Professor of Otology, University of Buffalo


### Acute and Chronic Middle-Ear Suppuration, With Special Reference to Treatment.[1]

By GEORGE F. COTT, M. D.,
Clinical Professor of Otology, University of Buffalo.

THIS title refers of course to the middle-ear only, but as the external canal is frequently affected it might be proper to call attention to this condition as well. The external canal may be affected with boils or furuncles; they inhabit the parts generally situated near the external margin of the meatus and are extremely painful; the canal, especially if narrow, may be closed entirely or nearly so. Then chronic eczema may cause so much thickening that the drumhead cannot be examined for some time. The skin and periosteum of the external canal are closely united; any infection, which elsewhere in the body would be styled cellulitis, would here become periostitis, with excruciating pain and consequent swelling. Neuralgia of the fifth pair sometimes produces swelling of the auricle and canal, with pain, simulating middle-ear disease. Excepting eczema these conditions and many others, acute and chronic, are found with or without middle-ear inflammation.

### FREQUENCY OF MIDDLE-EAR INFLAMMATION.

Adolescence seems to be the most fertile period; many cases, however, occur in early childhood following exanthematous diseases, but most cases occur as a result of vegetations in the nasopharynx, commonly called adenoids. Since the advent of influenza the number has been much augmented. Dr. Wyatt Wingrave, of London, recently reported to the Otological Society of the United Kingdom on 100 cases of middle-ear suppuration, acute and chronic, as they occurred in the Central London Throat Hospital. Most cases occurred from 15 to 21 years, the least, under one year, and over 50. Forty-two patients had a history of tubercle or clinical evidence of such in the patient, independently of the ear. Twenty-four had tubercle bacilli and seventeen more had permanent tuberculosis.

This finding was by a specialist; the general practitioner however would, if he looked for them, find many which the specialist never sees. In order to bring this more forcibly to your attention, I will mention that H. N. Leaven, in *American Practitioner and News* for January, 1903, quotes Panfi as having found in 100 postmortem examinations in children under three years of age who died from acute or chronic disease, in the majority of which otitis

1. Read at the Geneva Medical Society, September, 1904.

media was not suspected, all but nine had middle-ear inflammation and of these 73 were bilateral. Barth, of Leipzig, Germany, observed 600 infants and found in 80 per cent. otitis media present. These statements, made by most competent observers should make us more cautious in the examination of children suffering from infectious diseases, for it is very difficult to prove that middle-ear inflammation arises within that cavity.

### PECULIAR IMMEDIATE RESULTS.

After pain has lasted from four hours to several days, the drumhead ruptures and there is a flow of serum or pus which may cease after a longer or shorter period, or may persist indefinitely, causing a great deal of destruction within the drum, impairing hearing, due to thickening of tissue, interfering with vibratory motion, or affecting the receptive apparatus or the internal ear. Occasionally the drumhead does not rupture, infection passing out of the middle ear by way of the periosteum and producing a mastoid periostitis simulating mastoiditis; or cells sometimes present, leading from the tympanum directly to the mastoid, may be affected without a channel leading to the antrum; commonly, however, the antrum is also involved. Pus may burrow through the mastoid process and down the side of the neck, forming abscess in the neck or anywhere lower down. In case a patient had repeated middle-ear suppuration in early childhood and each time recovered, apparently some slight inflammation of the throat may set up an acute ostitis of the temporal bone, and result in meningitis or sinus thrombosis. Brain abscess may develop in acute middle-ear suppuration, though rarely. Exfoliation of the cochlea in chronic cases has occurred and the patient recovered. The bone separating the middle-ear from the carotid artery, varying in thickness, has been involved, causing ulceration of the coats of the carotid and producing fatal hemorrhage. Twenty-one cases of this accident have been reported.

### PROGNOSIS.

Acute middle-ear suppuration in children, if properly treated, nearly always recovers with very little impaired hearing; if neglected, chronic suppuration commonly follows. In acute infectious diseases it is sometimes extremely difficult to check the discharge, but persistent efforts are generally crowned with success, not however until serious damage is done as regards hearing.

### SOME DANGER SYMPTOMS.

La grippe is the most formidable enemy the middle ear has and is perhaps the most difficult to get rid of. Persistent pain, deep seated, is invariably due to antral involvement in acute cases; in those of subacute or a chronic nature, pachymeningitis or extradural abscess. Irregular temperature, mostly subnormal, with slow pulse, chills or numerous chilly sensations, slow cerebration and headache will direct attention to brain abscess. Steeple-peaked temperature, varying from 2 to 9 degrees in 12, 24 or more hours during middle-ear inflammation shows involvement of one of the sinuses, probably the lateral or sigmoid; bulging of the eye or swelling of the soft parts may mean involvement of the circular sinus. Bulging of the external ear, with or without pain, is invariably mastoid periostitis, with infiltration sometimes to the extent of an inch in thickness, but this periostitis may be present without immediate middle-ear involvement.

TREATMENT.

In acute cases pain must be relieved at all hazards. Hot sterile salt solution injected into the external canal with a fountain syringe four or five times a day, and hot applications over the ear and mastoid process often give great relief. Antiphlogistine may first be applied behind the ear; this substance is composed of pipe-clay and glycerine properly mixed. If the drumhead is found reddened and swollen, paracentesis will often give prompt relief. In the external canal periostitis or furunculosis incision may be called for, but is very painful; it may be relieved at times by the introduction of equal parts of anilin oil and alcohol and 5 per cent. of cocaine. In this condition of the middle-ear inflammation relief is often obtained by inserting into the external canal a bougie recommended by Dr. George L. Richards, of Fall River, Mass., consisting of:

| | |
|---|---|
| Carbolic acid | 1-6 grain |
| Fl. ext. opium | 1-4 grain |
| Cocaine | 1-14 grain |
| Atropin | 1-30 grain |
| Water | 52 minims |
| Gelatin | 18 grains |
| Glycerin | 158 grains |

To make 42 bougies.

After the acute symptoms have passed off the ear may be frequently dried with a cotton swab and some absorbing powder blown into the canal, but if there be periostitis, Wild's incision will be sufficient. If the mastoid is affected, chiseling away the broken down cells will become necessary; if the antrum is also involved this likewise must be opened. In chronic suppuration, which is always caused by caries of some part of the middle ear, more thorough measures become necessary, such as the Stacke modified radical, or radical operation.

In uncomplicated cases the conservative treatment is to be preferred. This is within the reach of every practitioner, and if carried out carefully will give good results in the vast majority of cases. Impaired hearing will depend generally upon the amount of thickening in the middle, or involvement of the internal ear; very often however it is good even though no vestige of the drumhead or ossicles remain. The important treatment to the practitioner is that which he can carry out in his office. When granulation polypi are present they must be removed which, generally, is not painful even without cocaine. Syringing is often useless; still, in exceptional cases good results follow. The middle ear is best cleansed with a little peroxide of hydrogen, after which the interior should be dried thoroughly and henceforth the dry method should be used exclusively.

I will not mention all the powders used or liquids instilled, generally by the patient at home until he gives up in disgust and does as the older doctors recommended, outgrow it. We are beyond that period now and get results by medicinal or surgical means. To outgrow it, is a most dangerous procedure. In my last five radical operations, out of a total of 45, I found localised pachymeningitis in two, epidural abscess in another, septic sinus thrombosis in a third, and brain abscess in the fourth. When we have none of these complications present, but simply limited caries, the practitioner can treat the case quite as well as the surgeon. This may be done by cleaning the ear, then applying hot air through a conducting pipe of hard rubber, introducing it an inch into the meatus so that all the heat will be expended in the middle-ear. This may be done every day at the office or at home and used 5 to 15 minutes at a sitting. The odor will disappear after a very few treatments and the ear gradually become dry.

Some cases however are not so markedly influenced, and in children the hot air cannot be applied; hence in such cases we must use other means. The most efficient I have found to be pyoktanin. A small spindle-shaped piece of cotton is dipped into the powder and introduced deep into the meatus through a speculum; the meatus is then plugged with absorbent cotton, which is left for two days and then removed. The canal should be dried with a swab and another plug introduced, and so on until the discharge ceases entirely. It may require several weeks or months to accomplish the desired result, but persistency will often overcome the need of surgical intervention.

The formula introduced by Dr. Holbrook Curtiss, of New York, and practised by Dr. George L. Richards, of Fall River, is made of pyoktanin, one part; boracic acid impalpable powder, nine parts, which are triturated. One ounce of this powder will last several years. The only care necessary is to prevent the purple color from staining the skin outside the meatus; it can, however, be removed with peroxide of hydrogen or preferably with alcohol. In many cases where, for various reasons, pyoktanin cannot be used, the following often gives excellent results: 5 per cent. resorcin in one hundred parts of alcohol, to be dropped into the ear daily or twice a day.

In acute cases it may be well to apply leeches to influence the blood supply to the parts affected. The internal maxillary artery enters the external auditory canal anteriorly; in inflammation of the canal the leech should be applied in front of the ear. In affection of the middle ear, which is supplied by the styloid artery and which courses behind the ear, the leech is applied over the mastoid process, taking care to plug the external meatus to prevent the leech from entering it. Sometimes, when the opening in the drumhead is small, it may be well to remove a part of it so that treatment can be more readily applied to the middle ear, and when the attic seems to be the source of the trouble this part particularly must be syringed out and then kept dry.

To recapitulate: in acute middle-ear inflammation with suppuration employ frequent hot saline injections, with drying powders. When in great pain, paracentesis and before this possibly the anodyne bougie, leeches, poultices or other hot applications.

In chronic suppuration, keep the middle ear dry, removing polypi or granulations with a snare or sharp spoon; then apply super-heated air every other day, followed by a powdered pyoktanin cotton plug introduced into the middle ear.

In chronic eczema, which is always due to the irritating discharge, keep the parts thoroughly dry, then apply 5 per cent. argyrol, which dries quickly, and afterward resorcin ointment. The most obstinate case will yield to this simple treatment, and after improvement is perceptible the ointment alone should be used.

The hot air apparatus which I use can readily be constructed from material obtained in the shops and is inexpensive. The more expensive outfits I have found cumbersome and more or less useless.

85 North Pearl Street.

---

Application of the Idea.—Gayman (in front of the mirror) —I don't know whether to wear a white necktie or a black one this evening. What is good form for a man over sixty?

Mrs. Gayman—Chloroform.—*Chicago Tribune.*

